# Deformable registration using edge‐preserving scale space for adaptive image‐guided radiation therapy

**DOI:** 10.1120/jacmp.v12i4.3527

**Published:** 2011-11-15

**Authors:** Dengwang Li, Hongjun Wang, Yong Yin, Xiuying Wang

**Affiliations:** ^1^ College of Physics and Electronics Shandong Normal University Ji'nan 250014 China; ^2^ School of Information Science and Engineering Shandong University Jinan 250100 China; ^3^ Department of Radiation Oncology Shandong Cancer Hospital Jinan 250117 China; ^4^ Biomedical & Multimedia Information Technology Research Group University of Sydney Australia

**Keywords:** cone‐beam CT, deformable registration, edge‐preserving scale space, multiscale image registration, adaptive image‐guided radiation therapy

## Abstract

Incorporating of daily cone‐beam computer tomography (CBCT) image into online radiation therapy process can achieve adaptive image‐guided radiation therapy (AIGRT). Registration of planning CT (PCT) and daily CBCT are the key issues in this process. In our work, a new multiscale deformable registration method is proposed by combining edge‐preserving scale space with the multilevel free‐form deformation (FFD) grids for CBCT‐based AIGRT system. The edge‐preserving scale space, which is able to select edges and contours of images according to their geometric size, is derived from the total variation model with the L1 norm (TV‐L1). At each scale, despite the noise and contrast resolution differences between the PCT and CBCT, the selected edges and contours are sufficiently strong to drive the deformation using the FFD grid, and the edge‐preserving property ensures more meaningful spatial information for mutual information (MI)‐based registration. At last, the deformation fields are gained by a coarse to fine manner. Furthermore, in consideration of clinical application we designed an optimal estimation of the TV‐L1 parameters by minimizing the defined offset function for automated registration.

Six types of patients are studied in our work, including rectum, prostate, lung, H&N (head and neck), breast, and chest cancer patients. The experiment results demonstrate the significance of the proposed method both quantitatively with ground truth known and qualitatively with ground truth unknown. The applications for AIGRT, including adaptive deformable recontouring and redosing, and DVH (dose volume histogram) analysis in the course of radiation therapy are also studied.

PACS numbers: 87.57.Gg, 87.57.Ce, 87.62.+n

## I. INTRODUCTION

Radiation therapy planning is currently limited to a single three‐dimensional (3D) anatomical CT image at the onset of treatment. This idea may result in severe treatment uncertainties, including the irradiation of risk organs and reduced tumor coverage.^(^ ^1^ ^)^ It is necessary to incorporate daily images into treatment process for patient setup and treatment evaluation.^(^ ^2^ ^)^ Recently, the advancement of volumetric imaging in daily treatment room by using KV (kilo‐voltage) CBCT (cone‐beam computer tomography) has provided the imaging data needed to perform AIGRT (adaptive image‐guided radiation therapy).^(^ ^3^ ^)^ The concepts of AIGRT provide methods to monitor and adjust the treatments to accommodate the changing and moving of the patient. Ideally, AIGRT is implemented where the patient alignment and radiation beam angles are continuously updated to maximize the radiation dose to the tumor and minimize radiation to healthy organs in the treatment room,^(^ ^4^ ^)^ and it is possible that the treatment is evaluated periodically and the plan for radiation therapy is adaptively modified for the remaining course of radiotherapy.

CBCT‐based AIGRT system is typically implemented in the following way:^(^ ^4^ ^–^ ^6^ ^)^ planning CT (PCT) images are obtained several days or weeks before the treatment. Then radiation planning is done including tumor and organ contouring, radiation beam optimization, and dose volume analysis. Just before the treatment, CBCT images are obtained in the treatment room and are used to register with PCT for adjusting the treatment parameters, which can maximize the radiation dose delivered to the tumor and minimize radiation to the healthy tissue. This enables the oncologists to adjust the treatment plan to account for patient movement, tumor growth, and deformation of the surrounding tissues. Hence, this treatment program would require real‐time deformable registration algorithm for PCT and daily CBCT images obtained during every intrafraction treatment.

Deformable registration for PCT and CBCT has been investigated by several literatures. B‐spline–based registration algorithms are used for estimating the deformation fields as shown in Paquin et al.,^(^ ^5^ ^)^ where multiscale technique is used for efficiency. In Brock et al.,^(^ ^7^ ^)^ finite element model (FEM) is used for getting more accurate assessment of tumor response. Demons registration method is also used for CBCT‐guided procedures in the head and neck cancer radiation therapy.^(^ ^8^ ^)^ Optical flow‐based deformable registration is also used for CBCT‐based IGRT; in Ostergaard Noe et al.,^(^ ^9^ ^)^ both CBCT to CBCT and CBCT to PCT registration are performed, and the acceleration obtained using GPU (graphics programming unit) hardware makes it possible for the registration to be done online for the CBCT system. Deformable registration methods for CBCT‐based AIGRT system mentioned above can be classified into physical model‐based methods described by partial differential equations of continuum mechanics, and basis function expansions‐based methods derived from interpolation and approximation theory according to transformation models.^(^ ^10^ ^)^ Physical model‐based methods have the advantage of providing physically realistic solutions. However, solving the Navier PDEs (partial differential equations), which are the expressions of the physical model‐based methods, is particularly computationally intensive.^(^ ^10^ ^–^ ^11^ ^)^ The radial basis functions and piecewise polynomials (splines) based methods are using a set of basis functions, and the coefficients are adjusted so that the combination of basis functions fit the displacement field.^(^ ^10^ ^)^ Radial basis functions and piecewise polynomials (splines) based methods are widely used for medical image registration in recent years.^(^ ^5^ ^,^ ^10^ ^,^ ^12^ ^)^ Despite the significant progress which has been made in recent years, deformable registration algorithm for PCT and CBCT is still a hot research topic for clinical application and remains a challenging task.^(^ ^5^ ^,^ ^13^ ^)^

Furthermore, mutual information (MI) as a similarity metric has the advantage of directly exploiting the raw data without requiring segmentation or extensive user interaction, which is the most popular accepted intensity measure for registration, particularly for multimodal images.^(^ ^14^ ^–^ ^15^ ^)^ However, in addition to the high computational complexity of MI for 3D image, optimization process can always be trapped into local extremes when searching for the global optimum and, hence, result in misalignment. Although multiscale registration methods can improve the registration efficiency including speed, accuracy, and robustness,^(^ ^5^ ^,^ ^16^ ^)^ Pluim et al.^(^ ^17^ ^)^ believed that one reason for misregistration in MI‐based registration is the absence of spatial information in MI measurement.

In our work, aiming for reducing radiation therapy uncertainties and increasing replanning efficiency in CBCT‐based AIGRT system, we designed a new multiscale deformable registration framework that can improve registration accuracy and robustness for PCT and daily CBCT in the treatment process. Our framework is constructed by combining the edge‐preserving scale space with the coarse–to–fine FFD grids. The edge‐preserving scale space, which is able to select edges and contours of the images according to their geometric size, is derived from the total variation model with the L1 norm (TV‐L1). At each scale, despite the noise and contrast resolution differences between the PCT and CBCT, the selected edges and contours are sufficiently strong to drive the deformation using the FFD grid, and the edge‐preserving property ensures more meaningful spatial information for MI‐based registration. Finally, the deformation fields are gained by a coarse to fine manner. Experiment results and clinical applications demonstrate the efficiency of the proposed method.

## II. MATERIALS AND METHODS

### A. Registration method

#### A.1 Hierarchical multiscale decomposition based on TV‐L1 scale space

The proposed multiscale registration framework is based on edge‐preserving scale space which is derived from the total variation with the L1 norm.^(^ ^18^ ^)^ TV‐L1 model has been successfully used in image processing, with the benefit of edge preserving and unique edge and contour selecting property, for instance, in the area of face recognition,^(^ ^19^ ^)^ DNA microarray data analysis,^(^ ^20^ ^)^ and other image processing.^(^ ^21^ ^)^ Here we give the theory analytical properties for TV‐L1 scale space.

Based on the work of Chan and Esedoglu,^(^ ^18^ ^)^ within TV‐L1 model the input image $I_{0}$ can be modeled as the sum of the image cartoon *I* and texture $V(V(x)=I_{0}(x)-I(x))$. The image cartoons contain important contours and edges which can provide meaningful spatial information for MI‐based registration. The rest of the image, which is texture, is characterized by irregular components and noise. Formally, the TV‐L1 model can be formulated using the following energy function:

(1) $$E(I,\lambda )=\underset{I}{\min}\int_{\Omega }|\nabla I(x)|dx+\lambda |I_{0}(x)|dx$$

It has been proved that solving energy function Eq. (1) is equivalent to solving the following level set based geometrical problem:^(^ ^18^ ^)^

(2) $$E(I,\lambda )=\underset{I}{\min}\int_{-\infty }^{+\infty }Per(\{x:I(x)>I\})+\lambda Vol(\{x:I(x)>I\}⊕\{x:I_{0}(x)>I\})dI$$

In Eq. (2), *Per(.)* is the perimeter and *Vol(.)* is the volume; and for any set S1 (Set 1) and S2 (Set 2), S1⊕S2:=(S1∪S2)−(S1∩S2) where ‘:=’ means ‘define’, ‘U’ means ‘union’, ‘∩’ means ‘intersection’, and ‘−’ means ‘exclude’. By using Eq. (2), we can get the following geometric properties of the solution to Eq. (1):^(^ ^18^ ^)^

- Given a disk image $I_{0}(x)=c_{1}1_{B_{r(y)}}(x)$, image with the intensity $c_{1}$ in the disk $B_{r}(y)$ which is centered at *y* and with radius *r*, and with the intensity *0* anywhere else, then we have
  (3) $$I(x,\lambda )=\{\begin{array}{l} 0;(0\leq \lambda \leq 2/r)\\ \left\{s1_{B_{r}(y)}(x):0\leq s\leq c_{1}\};(\lambda =2/r\right)\\ c_{1}1_{B_{r}(y)}(x);(\lambda >2/r) \end{array}$$
- Given $I_{0}=c_{1}1_{B_{r1}(y_{1})}(x)+c_{2}1_{B_{r2}(y_{2})}(x)$, where $0<r_{2}<r_{1}$ and $c_{1},c_{2}>0$, then we have
  (4) $$I(x,\lambda )=\{\begin{array}{l} 0;(0<\lambda <2/r_{1})\\ c_{1}1_{B_{r_{1}}(y)}(x);(2/r_{1}<\lambda <2/r_{2})\\ \left(c_{1}1_{B_{r_{1}}(y)}+c_{2}1_{B_{r_{2}}(y)})(x);(\lambda >2/r_{2}\right) \end{array}$$

**Figure 1:**
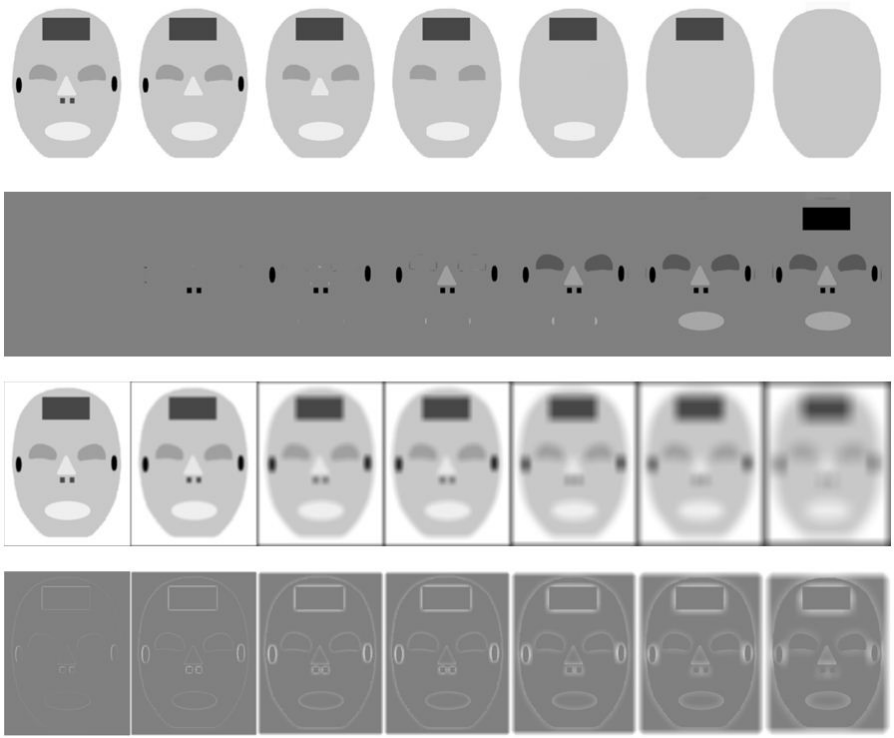
The first row are multiscale decomposition images using TV‐L1 scale space filtering with λ=0.26, 0.17, 0.1, 0.08, 0.06, 0.04, and the first image is the original image; the second row shows the difference between original image and TV‐L1 decomposition images; the third row shows multiscale decomposition images using Gaussian scale space with standard deviation σ=2, 4, 8, 16, 32, 64, 80; and the fourth row shows the differences between original image and Gaussian decomposition images.

Clinical medical images can be thought of as composed of different organs and tissues which are different from each other by geometric sizes. Suppose that medical image I is composed by n components with different geometric sizes, as illustrated in Fig. 1. It can be formulated as:

(5) $$I=\sum_{i=1}^{n}C_{i}$$

where the components are classified according to their geometric sizes, and $C_{1}>C_{2}>C_{3}.\ldots .>C_{n}$. Normally, considering noise existing in medical images, the smallest pattern $C_{n}$ can be considered as noise in image I which can also be removed by TV‐L1 scale space filtering. Based on Eqs. (3) and (4), TV‐L1 can be used for scale space decomposition of medical images, and features of different sizes in medical images can be extracted from I by applying different values of λ. This λ is in inverse proportion to the geometric size of the different component $C_{i}$.

The images are then decomposed with multiscale representation, the minimization of energy function E(I, λ) results in a decomposition $I_{0}=I(\lambda )+V(\lambda )$, where I(λ) extracts the edges and contours of $I_{0}$, and V(λ) extracts the textures or noise at scale λ. This interpretation depends on the scale λ, since edges and contours at scale λ consist of edges and contours when viewed under a refined scale (e.g., $\lambda_{1}$ where $\lambda_{1}<\lambda$). Thus we can do hierarchical multiscale decomposition of $I_{0}$ by repeating this process following Eq. (6). Starting with an initial scale $\lambda =\lambda_{1}$, we obtain the multiscale decomposition of the image $I_{0}$:

(6) $$\begin{array}{l} I_{0}=I(\lambda_{1})+V(\lambda_{1});\;\;\;[I(\lambda_{1}),V(\lambda_{1})]=E(I_{0},\lambda_{1});\;\;\;\lambda_{1}>0\\ I(\lambda_{1})=I(\lambda_{2})+V(\lambda_{2});\;\;\;[I(\lambda_{2}),V(\lambda_{2})]=E(I(\lambda_{1}),\lambda_{2});\;\;\;\;\lambda_{1}>\lambda_{2}\\ I(\lambda_{2})=I(\lambda_{3})+V(\lambda_{3});\;\;\;[I(\lambda_{3}),V(\lambda_{3})]=E(I(\lambda_{2}),\lambda_{3});\;\;\;\;\lambda_{2}>\lambda_{3}\\ I(\lambda_{n-1})=I(\lambda_{n})+V(\lambda_{n});\;\;\;[I(\lambda_{n}),V(\lambda_{n})]=E(I(\lambda_{n-1}),\lambda_{n});\;\;\;\;\lambda_{n-1}>\lambda_{n} \end{array}$$

**Figure 2:**
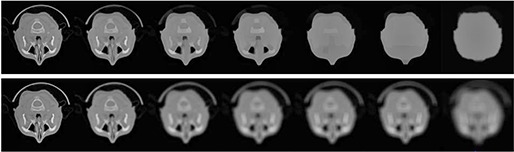
Multiscale decomposition using TV‐L1 scale space filtering in row 1 with λ=0.7,0.45,0.3,0.2,0.15,0.12 and Gaussian scale space filtering in row 2 for CT image with σ=2,4,8,16,32,64, and 80.

Now we get n‐level multiscale decomposition of $I_{0}$ as Eq. (6). Furthermore, the geometric properties of TV‐L1 decomposition developed for 2D images can be extended to 3D images straightforwardly.^(^ ^18^ ^)^ The TV‐L1 scale space is capable of selecting edges and contours of an image according to their geometric sizes rather than intensities with the merit of edge preserving property. This capability is illustrated in row 1 of Fig. 1 and in row 1 of Fig. 2, which shows that in larger scales (low resolution) edges and contours of smaller components can be removed by adjusting λ, whilst edges and contours of larger regions are kept in the corresponding scale. Comparatively, because the smoothness is isotropic in traditional Gaussian scale space filtering, all edges and contours are blurred at coarse Gaussian scales, as illustrated in row 3 of Fig. 1 and in row 2 of Fig. 2.

From Figs. 1 and 2, comparing with the Gaussian scale space, TV‐L1 scale space shows advantages of edge and contour selecting with the merit of edge preserving property. This multiscale decomposition can ensure more meaningful spatial information for MI‐based registration where the selected edges and contours are sufficiently strong to drive the deformation by a coarse to fine manner.

There are several numerical methods for solving the TV‐L1 energy function. The most classic one is the time marching PDE method based upon Euler‐Lagrange equations.^(^ ^18^ ^)^ Other methods include second order cone programming method,^(^ ^22^ ^)^ and parametric max‐flow algorithms.^(^ ^23^ ^)^ In our work, we use the parametric max‐flow method to solve the TV‐L1 model for efficiency.

### B. Multiscale deformable registration algorithm

#### B.1. B‐spline–based FFD deformable registration

Because the proposed method mainly focused on the multiscale strategy for improving the efficiency, in different scales we can use different deformable models for computation efficiency. Conventional methods always use affine or rigid transformation model in coarser scales for simplification, and then use more advanced deformable models in finer scales.^(^ ^16^ ^)^ However, deformable models with little computation in coarse scale can improve the registration performance. Many researches argue that B‐splines are optimal as approximating functions for registration.^(^ ^10^ ^)^ Furthermore, these basic functions can be extended to multivariate ones using tensor products. The FFD is the most frequently used one for medical image registration.^(^ ^10^ ^)^ B‐spline–based free‐form deformation (FFD) registration method has the advantage over other spline‐based deformation models (e.g., TPS (thin plate splines)) in that perturbing the position of one control point only affects the deformation in a neighborhood of that point.^(^ ^10^ ^)^ Changing one control point only affects the deformation in a local neighborhood. The control points act as parameters of the B‐spline deformation model, and the freedom degree of deformation field depends on the resolution of the mesh of control points. A coarse spacing of control points can model global deformation, while a fine spacing of control points can model local deformations.^(^ ^24^ ^)^ Furthermore, the number of control points determines the degrees of freedom in deformation model and the computational complexity. So coarse–to–fine multilevel FFD grids can be conveniently combined with the multiscale edge‐preserving scale space for registration. In lower image scale, coarse spacing of control points are used for estimating the global deformation, while in higher image scale, fine spacing of control points are used for estimating the local deformation. In our work, multiscale FFD grids of control points were chosen automatically, and there is a trade‐off between the model flexibility and its computational complexity.

#### B.2. Multiscale registration by combining edge‐preserving scale space with multilevel FFD grid

**Figure 3:**
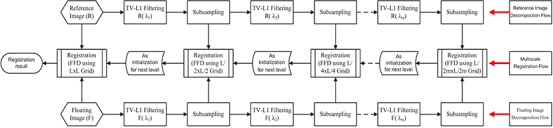
Diagram of the proposed multiscale registration algorithm. The first stage is the multiscale decomposition stage which is illustrated from left to right (in the first row and the third row), and the second stage is the multiscale deformable registration process which is illustrated from right to left (in the second row).

In the proposed method (shown in Fig. 3), at stage 1 the reference image and the floating image are first decomposed with a multiscale representation using TV‐L1 scale space filtering (the first row and the third row in Fig. 3). The scale levels are denoted as $L_{1}\ldots L_{m}$, where *m* is the number of multiscale levels.

At stage 2, the decomposed image pairs are registered using proposed multiscale registration framework in coarse‐to‐fine manner (the second row in Fig. 3). Because stage 2 uses the intermediate results from stage 1, so the flowchart is first from left to right for multiscale decomposition, and then from right to left for multilevel registration (Fig. 3). The deformation is described by FFD based on B‐splines. The control points act as parameters of FFD and the degree of deformation is in dependant on the resolution of the mesh of control points. A large spacing of control points allows modeling of global deformations, while a small spacing of control points allows modeling of highly local deformations.

As illustrated in Fig. 3, the coarsest scale images in TV‐L1 scale space — which include the largest edges and contours of original images by removing the smaller patterns and noises from the original image — are first utilized for an initial registration. Once the registration has finished with the low resolution grids, it then proceeds to initialize higher resolution.

Because only global structures remain in the coarsest scale, the coarsest FFD grid is used for estimating the global deformation with a rapid convergence. The optimum set of transformation parameters at a coarser resolution level is up‐sampled to become the starting point for the next finer resolution level with higher resolution FFD grids. The registration can then be optimized by gradually introducing more detailed components at finer scale images. At each scale, the selected edges and contours are sufficiently strong to drive the deformation, which makes the algorithm more efficient in accuracy and robustness. Consequently, the local deformation is gradually gained through coarse‐to‐fine manner.

Because the size of the overlapping part of the image pairs influences the MI measure, normalized measure of MI which is less sensitive to changes in overlap is used for registration. In our work, normalized mutual information (NMI) as in Studholme et al.,^(^ ^25^ ^)^ which can give promising accuracy and robustness for registration, is used as similarity measure. Our multiscale registration can accelerate NMI computation process by coarse to fine manner without decrease accuracy and robustness. In order to further improve computational efficiency at each successive level of the framework, TV‐L1 scale space filtering is preceded by equidistant subsampling to reduce the image size, as well as susceptibility to noise. So the proposed framework can combine edge preserving and scale selection properties of the TV‐L1 with the FFD grids, which can provide more meaningful spatial information for NMI based registration while maintaining the high efficiency of the pyramid framework. The NMI used in our work is defined as follows:^(^ ^25^ ^)^

(7) $$\text{NMI}(A;B)=\frac{H(A)+H(B)}{H(A,B)}$$

where *H(A)* is the Shannon entropy of the image A, and *H(A,B)* is the joint entropy of image A and image B.

#### B.3. Estimation of the optimal TV‐L1 parameter for automated registration

As explained above in Eqs. (1) and (4), the choice of parameter λ determines the structures that are kept in each scale, and proper estimation of λ is important for building the multiscale registration framework. The selection of λ is related to image properties, and is often determined heuristically and experimentally in image processing.^(^ ^18^ ^–^ ^19^ ^,^ ^22^ ^–^ ^23^ ^)^ To achieve automated registration for clinical applications, estimation of the optimal value for TV‐L1 model is proposed by training and minimizing an offset, which is defined as:

(8) $$Offset=\sum_{n}\kappa_{n}\left|\rho_{n}^{\prime }-\rho_{n}\right|$$

where *n* is the number of transformation parameters, $\rho_{n}$ are the ground truth parameters of transformation between the reference and floating image, $\rho_{n}\prime$ are the transformation parameters obtained by the registration algorithm, and $K_{n}$ are weighting coefficients.

**Figure 4:**
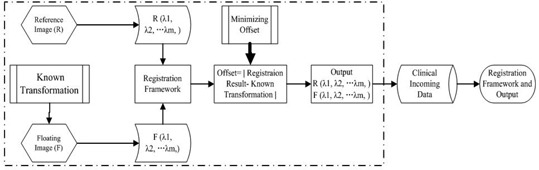
Diagram of automated estimating optimal λ (m level).

For deformable registration, the transformation freedom is always innumerable. From Eqs. (3) and (4), λ is in inverse proportion to the geometric size of the different components in the image. Suppose that λ is the same when it is optimal for any freedom. In clinical application, we can then obtain an optimal λ by optimizing the simple rigid transformation process. Rigid transformation considers three translation parameters and three rotation parameters for 3D registration. Suppose that the influence of the transformation along each 3D direction on the offset is the same, and that the offset caused by 1° in rotation direction is equal to the offset caused by 1 mm in translation direction. In our estimation framework, the unit for the rotation parameter 8 is not degrees but radians; therefore, we should change radians into degrees for computation, and the weighting coefficients can be set as $k_{1}=k_{2}=k_{3}=1$ and $k_{4}=k_{5}=k_{6}=180/\varphi$. As shown in Fig. 4, λ can be obtained automatically by the following function:

(9) $$\underset{\lambda (\lambda_{R}^{1},\lambda_{R}^{2},\ldots \lambda_{R}^{m};\lambda_{T}^{1},\lambda_{T}^{2},\ldots \lambda_{T}^{m})}{MIN(Offset)}=MIN\left[\begin{array}{l} \left|x^{\prime }-x\right|+\left|y^{\prime }-y\right|+\left|z^{\prime }-z\right|\\ +180/\pi (\left|\theta_{1}^{\prime }-\theta_{1}\right|+\left|\theta_{2}^{\prime }-\theta_{2}\right|+\left|\theta_{3}^{\prime }-\theta_{3}\right|) \end{array}\right]$$

In Fig. 4, known transformations (KT) which can serve as ground truth are implanted on the floating images. In our experiment, well‐registered image pairs from hospital are used for estimating the λ. Once the optimal λ is determined, it can be used for all incoming datasets of the same type and size as the training data.

So we do not need to optimize the λ for each patient. When the patient data are received, we first will find the λ in the computer memory that is optimal for the same type and same size computed before. Here the same type means that the received data are from same modality with the one in the memory, and the same size means that they both have the same organ size, such as lung, liver, heart, or other organ. In our experiment, for application efficiency, we supposed that if two patients are the similar size, they will have similar size of lung, liver, heart, or other organ. So in clinical application, we can only consider an incoming patient size which is the same as the patient in computer memory, and then we can use the same λ. If we cannot find the same size patient in our memory, we can get the optimal λ using rigid transformation by minimizing Eq. (9).

Actually, for clinical image registration in our experiment, the optimal λ is not the one which can always select the proper organs, such as lung or liver. However, it always gives a good edge protection in different scales within images, so different edges and contours with difference geometric sizes are preserved in different scales. These protected edges with optimal λ can make the MI maximization in that scale, and then this can result in a good registration performance.

## III. RESULTS & DISCUSSION

### A. Data collection

Data were collected from 30 patients treated under the on‐board imager (OBI cone‐beam CT, Varian Medical Systems, Palo Alto, CA), which has been in routine clinical use in Shandong Cancer Hospital. The OBI consists of a diagnostic X‐ray tube and a KV flat‐panel imager, both mounted on robotic arms and designed for three main functions: orthogonal radiographs for 3D patient setup, KV‐CBCT and real‐time tumor tracking, and fluoroscopy. Before treatment planning, each patient underwent a series of imaging studies including intravenous contrast planning CT imaging (Brilliance CT Big Bore 16, Philips Healthcare, Andover, MA). Then the GTV (gross tumor volume) and PTV (planned target volume) were delineated on every section of the planning CT scans by the radiation oncologists. Radiation physicists contoured the organs such as liver, external surface, spinal cord, kidneys, spleen, and stomach. These contours were reviewed and edited by the radiation oncologists.^(^ ^26^ ^)^ Finally, treatment planning is made with four components including RTstruct (radiotherapy structure), RTplan, RTdose and DVH (dose volume histogram) analysis. In each planning CT image, the dose distribution and dose volume histogram are calculated by the radiation oncologists using the planning system installed in Shandong Cancer Hospital. At the time of each delivered treatment fraction, a KV CBCT scan was obtained for the patient during normal breathing.

Of the 30 patients investigated here, five had rectum cancer, five had prostate cancer, five had lung cancer, five had head‐neck cancer, five had breast cancer, and five had chest cancer. The method used for radiotherapy is IMRT (intensity‐modulated radiotherapy), CRT (conformal radiotherapy) or IMAT (intensity‐modulated arc therapy). Table 1 is the description of patients for the experiments, including patient ID, cancer type, radiotherapy method, image resolution, and voxel resolution for PCT and CBCT.

**Table 1:** Description of patients for experiments.

			*Resolution for PCT*		*Resolution for CBCT*	
*Patient ID*	*Cancer*	*Radiotherapy*	*Image (p)*	$Voxel(mm^{3})$	*Image (p)*	$Voxel(mm^{3})$
1–5	Rectum	1,3 IMRT 4,5 CRT	512×512×96	1.02×1.02×3	384×384×64	1.17×1.17×2.5
6–10	Prostate	6–10 IMRT	512×512×88	1.12×1.12×3	384×384×64	1.17×1.17×2.5
11–15	Lung	11–15 CRT	512×512×104	0.99×0.99×3	384×384×64	1.17×1.17×2.5
16–20	H&N	16–20 IMRT	512×512×96	1.12×1.12×3	384×384×64	0.65×0.65×2.5
21–25	Breast	21–25 IMRT	512×512×96	1.07×1.07×3	384×384×64	1.17×1.17×2.5
26–30	Chest	26–28 CRT 29,30 IMAT	512×512×96	0.84×0.84×3	384×384×64	1.17×1.17×2.5

### B. Quantitative evaluation of the synthetic results

**Figure 5:**
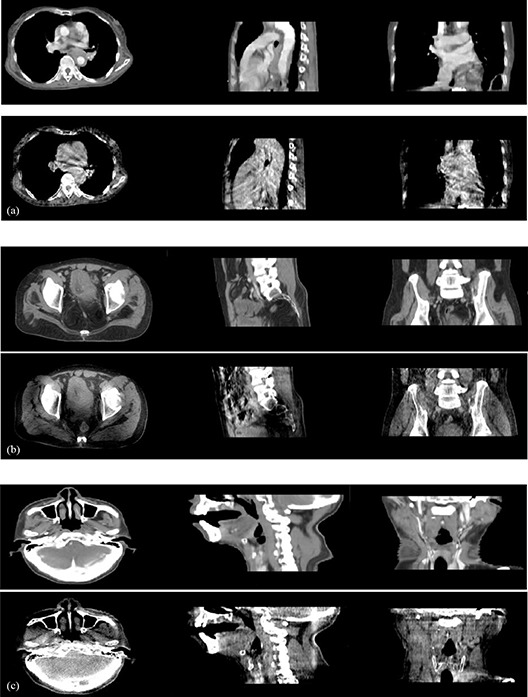
Planning CT and cone‐beam CT image comparisons, where images are 2D slices from 3D dataset: (a) chest images, (b) prostate images, (c) H&N images; in each group, the first row is CT image for planning, the second row is daily CBCT.

Generally, evaluating the performance of the deformable registration is a challenging task, because there is always lack of ground truth. In our work, for quantitatively evaluating proposed multiscale registration algorithm, we produce the registration problems in which the deformation between the reference images and floating images is known. Although same organ and tissue are displayed both in PCT and CBCT images, CBCT images contain low‐frequency components which are not present in PCT images (similar to inhomogeneity related components in magnetic resonance images) as illustrated in Fig. 5. So the main challenge in PCT‐CBCT image registration is thus accounting for the artifacts or noises that appear in one of them but not in another.

To simulate synthetic CBCT images using CT images, gray transformation (linear) and synthetic noise (both of Gaussian and salt–pepper noise) are added to the CT images, as shown in Eq. (10):^(^ ^16^ ^,^ ^27^ ^–^ ^28^ ^)^

(10) $$Image_{CBCT}=Gray\_Trans(Image_{CT})+Noise(Image_{CT})$$

**Figure 6:**
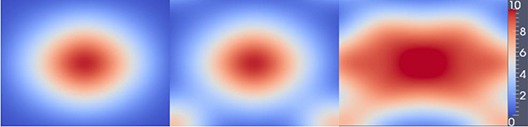
Three levels of deformation fields: from left to right, the deformation is from small to large and we index them using D1, D2, D3. The last one is meta‐image magnitude for the deformation, and the unit is the millimeter.

Then, we begin to deform the synthetic CBCT image which is derived from the corresponding CT image obtained by Eq. (10) using known deformations. We deformed the planning CT images using three defined splines vectors where the deformation fields are known. Three known splines vectors (Fig. 6) are D1 (deformation 1), D2, and D3. The source code for defined spline vectors used here for warping images is from open source software sponsored by the National Library of Medicine and the National Institutes of Health. Here D1, D2, and D3 are all two‐dimensional deformation fields; in our experiment, we deformed three‐dimensional images slice by slice using the two‐dimensional deformation fields. As illustrated in Fig. 6, the meta‐image magnitude tells us that the maximum shift of each case is 10 mm.

All 30 planning CT images that include six types of cancers are gray transformed and noise added for simulating CBCT images, and then three deformation fields are implemented on 30 planning CT images, respectively. Lastly, we register planning CT with the synthetic CBCT images.

To quantitatively evaluate accuracy of the registration algorithm, we compute the deformation difference (DD) with the sum of mean absolute difference between the deformation vector calculated by proposed method and the known defined vector deformation for each pair of images. The DD can be defined as:

(11) $$DD(C,K)=1/N\sum_{i=1}^{N}\left|W_{i}-K_{i}\right|$$

In Eq. (11), *N* is the total number of pixels, $W_{i}$ is the ith vector of the calculated deformation field, and $K_{i}$ is the ith vector of the known deformation field. Table 2 is the DD comparison between proposed edge‐preserving FFD registration method (EFFD) and normal B‐spline–based FFD method (NFFD).^(^ ^29^ ^)^ Each value in Table 2 is the mean value for five groups of PCT‐CBCT registration experiments using clinical data.

**Table 2:** The mean deformation difference comparison for the EFFD and NFFD (in millimeters).

	*H&N*	*Rectum*	*Prostate*	*Breast*	*Chest*	*Lung*
NFFD (D1)	0.504	0.553	0.554	0.606	0.615	0.703
EFFD (D1)	0.473	0.548	0.539	0.593	0.590	0.658
NFFD (D2)	0.530	0.569	0.563	0.625	0.673	0.725
EFFD (D2)	0.496	0.550	0.552	0.613	0.646	0.660
NFFD (D3)	0.581	0.594	0.595	0.714	0.723	0.753
EFFD (D3)	0.514	0.568	0.570	0.617	0.648	0.664

From Table 2, the deformation differences gained by EFFD are always smaller than the NFFD, which indicates that EFFD is more accurate than the NFFD for three levels of deformation. Furthermore, the registration accuracy is also affected by the deformation between reference image and floating image. Larger deformation is more difficult to resume than the small deformation. This is outlined in Table 2 by comparing D3, D2, and D1. This property is also informed from the different cancer types, including head and neck (H&N), rectum, prostate, breast, chest, and lung cancers. From Table 2, H&N case is the easiest one to recover the deformation, which is indicated by the smallest deformation difference. This is mainly accounted for by the smallest deformation in H&N case in clinical images, and in most cases, it can be considered rigid movement in clinical application. The rectum and prostate cases are in the same level when recovering the deformation, and the breast and chest are also in the same level to recover the deformation. The breast and chest cases are more difficult to recover the deformation than the H&N cases. The lung case is the most difficult type to recover the deformation and this is because the lung is the essential respiration organ in air‐breathing system, and it is always moving with the breathing all the time. The planning CT and CBCT for registration are always not in the same breathing phase because they are acquired at different time stages, which will result in the largest deformation comparing other organs.

Furthermore, from Table 2 we can conclude that EFFD is more robust than NFFD method with more stable lower DDs for six kinds of cancer registration and three levels of deformation. In particular when D3 deformation registration is combined with larger deformation organs including breast, chest, and lung cancers, the deformation difference will exceed 0.7 mm using NFFD, where the registration results have a low accuracy. However, EFFD can still get the stable registration results as in Table 2.

On the other hand, accuracy of the EFFD registration was also demonstrated by the mean MI comparison between reference and deformed floating image after registration, when using EFFD and NFFD, respectively, in Table 3. Corresponding to the previous deformation difference results, the mean MI is different from each other with different cancer types and different deformations. H&N case has the highest values, which indicates the most accurate results and the easiest cases to recover the deformation, and lung case has the lowest values which also indicates the most unreliable result and the most difficult to recover the deformation. Rectum, prostate, breast, and chest cases are between the H&N and lung cases. Similarly, each value in Table 3 is also the mean value for five groups of PCT‐CBCT registration experiments.

**Table 3:** The mean mutual information comparison for the EFFD and NFFD.

	*H&N*	*Rectum*	*Prostate*	*Breast*	*Chest*	*Lung*
FFD (D1)	0.894	0.815	0.818	0.737	0.728	0.685
EFFD (D1)	0.903	0.833	0.823	0.742	0.731	0.694
FFD (D2)	0.875	0.806	0.815	0.703	0.695	0.669
EFFD (D2)	0.896	0.814	0.819	0.716	0.704	0.675
FFD (D3)	0.842	0.798	0.810	0.689	0.686	0.643
EFFD (D3)	0.867	0.821	0.818	0.701	0.727	0.677

### C. Qualitative evaluation of the clinical results

**Figure 7:**
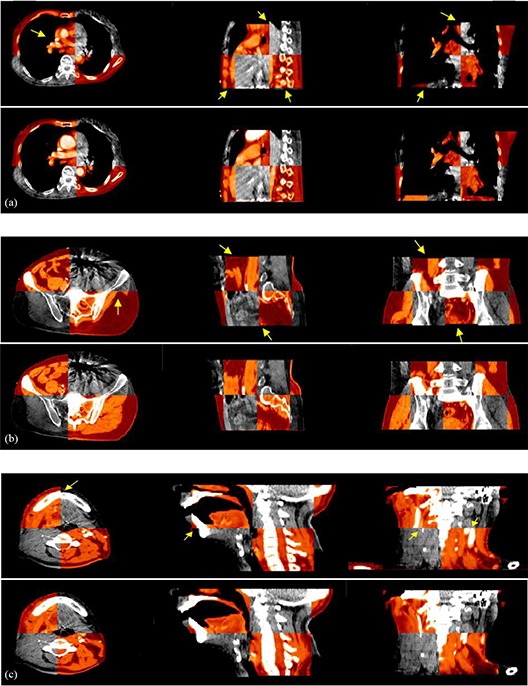
Checkerboards after planning CT and CBCT deformable registration, where images are 2D slices from 3D dataset: (a) result for chest images, (b) result for prostate images, (c) result for head and neck (H&N); in each group, the first row is using NFFD based method, the second row is using EFFD‐based method.

Next, we present the results obtained with the multiscale registration algorithm for clinical planning CT to CBCT, including lung, prostate, and H&N cancer patients as illustrated in Fig. 7. In each case, we illustrate a 2D slice of the 3D registration results from transversal, sagittal, and coronal directions, and checkerboards comparison after normal FFD registration method and our proposed edge‐preserving FFD registration method.

Here, we present the visual registration results for one lung example, one prostate example, and one H&N example. The results obtained with all other volumes are similar to those presented here. We have highlighted areas of misregistration in the checkerboard images after NFFD registration with arrows. In particular, we notice that misalignment always occurs in breast, chest, and lung cases, where larger deformation exists. However, we are able to recover this misalignment using proposed EFFD registration algorithm. The accurate registration of organ structures (where both small and larger deformations present in the registered pairs with proposed EFFD) is due to the fact that we combine edge‐preserving scale space with the free‐form deformation which can provide meaningful spatial information for the registration process. Furthermore, the advantage of our proposed method can also be demonstrated by higher MI from the registration results as outlined in Table 4.

**Table 4:** The mean mutual information comparison for the EFFD and NFFD.

	*H&N*	*Rectum*	*Prostate*	*Breast*	*Chest*	*Lung*
NFFD	0.883	0.807	0.812	0.732	0.724	0.673
EFFD	0.921	0.853	0.865	0.768	0.759	0.721

### D. Clinical application for AIGRT

After deformable registration of PCT and daily CBCT, we can do some applications with AIGRT by using the deformation fields. Adaptive deformable recontouring and redosing for improving treatment process are such applications. We performed qualitative evaluation of contours and doses generated automatically by visual inspection of the contour matches with the underlying structures in CBCT images. The deformation field provides voxel to voxel mapping between the reference image and the floating image.

**Figure 8:**
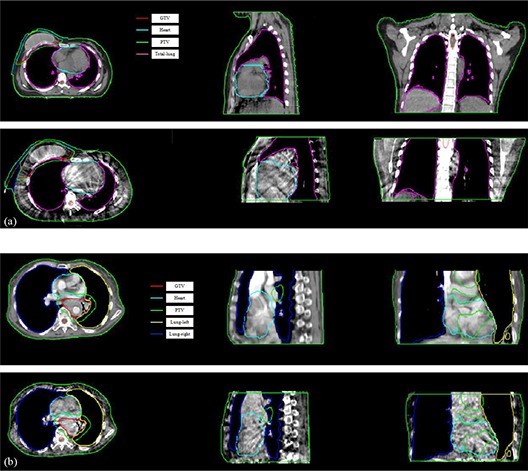
Adaptive deformable recontouring for daily CBCT images using the proposed registration algorithm, where images are 2D slices from 3D dataset. Group (a) is for the breast cancer patient, and group (b) is for the lung cancer patient. In each group, the first row is the planning CT which is contoured by the oncologist. The second row is automatically generated contours by the deformation map using proposed registration algorithm.

**Figure 9:**
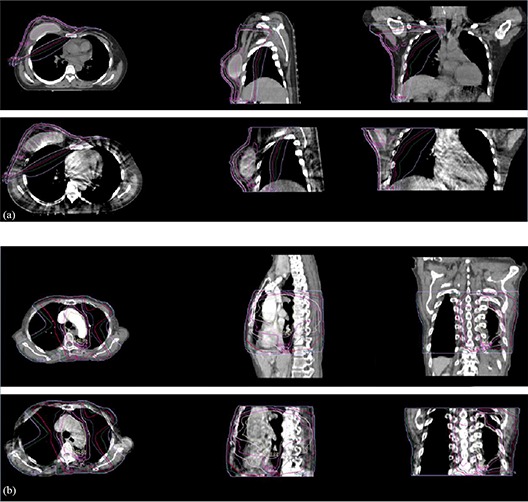
Adaptive deformable redosing for daily CBCT images using the proposed registration algorithm, where images are 2D slices from 3D dataset. Group (a) is for the breast cancer patient, and group (b) is for the lung cancer patient. In each group, the first row is dose distribution for the planning CT which is gained by the oncologist using planning system. The second row is automatically generated dose distribution by the deformation map using proposed registration algorithm.

The contours and doses in the floating PCT image can be transferred to the reference of CBCT image for replanning by the deformation maps. Figure 8 illustrates the adaptive deformable recontouring examples of the chest and lung patients where in planning CT images, normal organs, PTV, and GTV contours are delineated by radiation oncologists, while in daily CBCT images, normal organs, PTV, and GTV contours are generated automatically by the deformation map with proposed deformable registration algorithm. Similarly, Fig. 9 also illustrates adaptive deformable redosing with same chest and lung patients, where dose distribution in planning CT are calculated by the oncologists using the planning system, while corresponding dose distribution in CBCT is generated automatically by the deformation map using proposed deformable registration algorithm.

From Figs. 8 and 9, the contours and dose distribution are successfully transferred to the daily CBCT image from the planning CT by the proposed deformable registration algorithm (using the deformation maps). After statistic analysis by the radiation oncologists, the overlap between the automatically generated contours and the contours delineated by the oncologist using the planning system is on an average 95%, while the dose distribution overlap is also on an average 97%. The radiation oncologists concluded that the results from recontouring and redosing are helpful for clinical replanning using daily CBCT.

**Figure 10:**
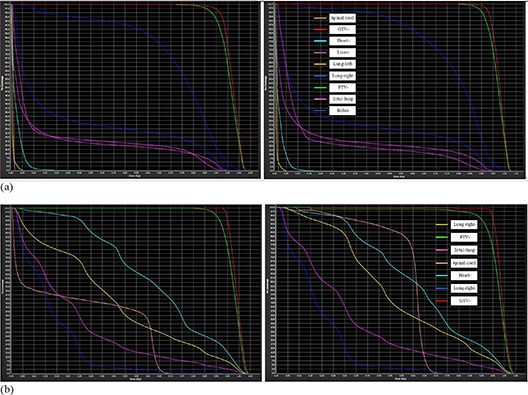
DVH analyzing (by normalized volume) for the daily CBCT after recontouring and redosing. Group (a) is for the breast cancer patient, and group (b) is for the lung cancer patient. In each group, the left one is DVH for the planning CT, which is gained by the oncologists using planning system. The right one is DVH, which is generated by automatically generated contours and dose distribution by the deformation map using proposed registration algorithm.

After obtaining the contours and dose distribution, DVH are analyzed for radiation therapy using the planning system (see Fig. 10). DVH for PCT are calculated by dose distribution which is generated by the treatment planning system, and DVH for CBCT are calculated by dose distribution which is gained by adaptive recontouring and redosing using proposed deformable registration algorithm. After analyzing by the oncologists and clinical doctors, the DVH for the CBCT is qualified for following radiation therapy and replanning.

During the experiments, the Insight Toolkit (ITK), which is an open source software sponsored by the National Library of Medicine and the National Institutes of Health, is used for building our B‐spline–based FFD framework. MATLAB (The MathWorks, Natick, MA) is used for TV‐L1 multiscale decomposition and parameter λ estimation. All the registration experiments are generated on HP Intel Duo core CPU T6570 with 2.10 GHz and RAM memory 2.00 GB. The computation time for the multiscale decomposition process with our 3D data requires approximately 80–100 sec per group of data, and the time for multiscale registration process requires approximately 180–210 sec per group of data. As we know, in an ideal AIGRT system, the planning CT and daily CBCT image will be registered in the treatment room where the whole process needs to be continuously updated. So currently we focus on improving the speed of our registration process in our future work, and parallel computing hardware can be used for the computational efficiency.

## IV. CONCLUSIONS

In our work, we proposed a new multiscale deformable registration method by combining edge‐preserving scale space with the multilevel free‐form deformation grids for CBCT based AIGRT system. The edge‐preserving scale space, which is able to select edges and contours of images according to their geometric size, is derived from the total variation model with the L1 norm. At each scale, despite of the noise and contrast resolution differences between the PCT and CBCT, the selected edges and contours are sufficiently strong to drive the deformation using the FFD grid, and the edge‐preserving property ensures more meaningful spatial information for mutual information‐based registration. Finally, the deformation fields are gained by a coarse to fine manner. Furthermore, considering clinical application we design an optimal estimation of the TV‐L1 parameters by minimizing the defined offset function for automated registration. In the experiments, six types of cancer patients are studied in our work, including rectum, prostate, lung, H&N (head and neck), breast, and chest. The experiment results demonstrate the significance of our proposed registration method. The applications of proposed deformable registration for AIGRT and ART, including adaptive deformable recontouring and redosing and DVH analysis are successfully implemented. The proposed deformable registration for planning CT and daily cone‐beam CT can be used for AIGRT, and the method proves to be an efficient tool to quickly transfer contours for radiation therapy treatment planning systems, and then aid following adaptive therapy.

However, as we know, CBCT has not only poor image contrast, but has severe artifact and low frequency components. All these make the registration process very difficult for obtaining accurate result in all cases. Actually, although our method can give better results in most normal cases compared with the conventional method, our method is weak in some instances. For example, our method is not effective when it comes to the case where the CBCT cannot show some of the organ or structure as in the planning CT. We have tried several cases, but they all failed. And these special cases have encourage us to research a more robust method for the critical clinical application.

Furthermore, images for registration of planning CT and CBCT are always not in the same breathing phase because they are acquired at different time stages. So the deformation for some cases is very large for the CT‐CBCT pairs. The research for the rule of lung breathing talks about the 4D CT direction^(^ ^30^ ^)^ and 4D CBCT direction.^(^ ^31^ ^)^ We can combine 4D CT technique for solving this problem so that we can make the planning CT and CBCT in the same phase for improving treatment accuracy.

## ACKNOWLEDGMENTS

The authors wound like to express thanks to the staff in the Department of Radiation Oncology, Shandong Cancer Hospital, and Institute, for their valuable suggestions to our work. This work is supported by the Shandong Natural Science Foundation (ZR2010HM010 and ZR2010HM071) and the NSFC (National Natural Science Foundation of China: 30870666).
